# Assessment of the anti-HBV like property of BEHP – a phthalate derivative and its *insilico* characterization

**DOI:** 10.1186/1471-2334-14-S3-E29

**Published:** 2014-05-27

**Authors:** AS Smiline Girija, V Duraipandiyan, K Pandi Suba, G Hariprasad, R Raghuraman

**Affiliations:** 1 Department of Microbiology, Meenakshi Ammal Dental College, Meenakshi University, Chennai, India; 2 Entomology Research Institute, Loyola College, Chennai, India; 3 Central Research Laboratory, Meenakshi Ammal Dental College, Meenakshi University, Chennai, India; 4 Department of Microbiology, Government Thoothukudi Medical College, Thoothukudi, India

## Background

Recent advances in the insilico virtual screening paves way towards efficient discovery of new drugs on the basis of drug target evaluation and analysis. The present study is thus an attempt to assess the anti-HBV like activity of Bis 2-(ethyl hexyl) phthalate [BEHP] from the black pigmented ink of South Indian squid, *Loligo duvauceli*.

## Methods

Fresh ink glands from *Loligo duvauceli* were dissected and the ink was subjected to crude solvent extraction. Chromatographic profiles were assessed by TLC and HPTLC. Fractionation of the bio active compounds was done by Silica gel column chromatography and the active fraction was subjected to GC-MS analysis. *In vitro* analysis of the anti-HBV like activity was assessed by pre incubating the HBV positive serum samples with the active fraction by performing ELISA. *In silico* virtual screening was analyzed with the latest bio informatics tools and databases.

## Results

TLC and HPTLC profiles yielded a compound with the Rf value of 0.76. The antimicrobial fraction upon GC-MS analysis revealed a chromatogram with nine peaks with BEHP possessing a highest percentage of area normalization (91%) with other few minor constituents. The ELISA results were negative for all the pre incubated HBV positive serum samples. Docking scores and the *in silico* analysis supported the preliminary screening results of ELISA and indicated the anti-HBV like activity of BEHP.

## Conclusion

This present study suggests that BEHP could be a novel anti-HBV agent and a promising candidate to combat viral hepatitis in near future with further clinical and toxicity evaluation.

